# Effect of Different Factors on Proliferation of Antler Cells, Cultured *In Vitro*

**DOI:** 10.1371/journal.pone.0018053

**Published:** 2011-03-28

**Authors:** Erika Kužmová, Luděk Bartoš, Radim Kotrba, George A. Bubenik

**Affiliations:** 1 Department of Ethology, Institute of Animal Science, Prague - Uhříněves, Czech Republic; 2 Department of Ecology, Charles University in Prague, Prague, Czech Republic; 3 Department of Integrative Biology, University of Guelph, Guelph, Ontario, Canada; The University of Hong Kong, Hong Kong

## Abstract

Antlers as a potential model for bone growth and development have become an object of rising interest. To elucidate processes explaining how antler growth is regulated, *in vitro* cultures have been established. However, until now, there has been no standard method to cultivate antler cells and *in vitro* results are often opposite to those reported *in vivo*. In addition, many factors which are often not taken into account under *in vitro* conditions may play an important role in the development of antler cells. In this study we investigated the effects of the antler growth stage, the male individuality, passaged versus primary cultures and the effect of foetal calf serum concentrations on proliferative potential of mixed antler cell cultures *in vitro*, derived from regenerating antlers of red deer males (*Cervus elaphus*). The proliferation potential of antler cells was measured by incorporation of ^3^H thymidine. Our results demonstrate that there is no significant effect of the antler growth stage, whereas male individuality and all other examined factors significantly affected antler cell proliferation. Furthermore, our results suggest that primary cultures may better represent *in vivo* conditions and processes occurring in regenerating antlers. In conclusion, before all main factors affecting antler cell proliferation *in vitro* will be satisfactorily investigated, results of *in vitro* studies focused on hormonal regulation of antler growth should be taken with extreme caution.

## Introduction

As the only completely regenerating organ found in mammals, deer antlers evoke rising interest of many scientists. Antlers can be used as an interesting and easily accessible model for bone growth processes as well as mammalian regeneration [1]–[3]. On the other hand, despite decades of being studied, a lot is still unknown about the regulation of antler growth. Various authors carried out *in vivo* and *in vitro* experiments and in many cases the correlations between antler growth and various hormones or growth factors, testosterone and IGF-1 in particular, and their effect on antler growth, are contradictory [1], [4]–[11]. As suggested earlier [8], [11], this inconsistency may lie in factors associated with the *in vitro* environment. Indeed, recently an increasing interest is paid to the influence of cultivation factors which can affect the proliferation and differentiation potential of cell cultures in vitro. This shows up especially for mesenchymal stem cell cultures [12]–[21]. Mesenchymal stem cells (MSC) were lately isolated also from pedicles and regenerating antlers of fallow deer [3]. Recently we confirmed that considerable amounts, up to 38% of these cells can be isolated from the regenerating antler tips of fallow and red deer, even though the amount of isolated MSC varied greatly depending on culture conditions [22].

Throughout the literature, experiments using pedicle [4], [5] or antler cells [9]–[11], [23], cells from different stages of antler development and growth, cultivated either as primary cultures [23] or after two passages [4], [5], [9]–[11], grown in medium containing foetal calf serum (FCS) [4], [5], [9]–[11], [23] or partially cultured in serum free conditions [4], [5], [9], [10] have been reported. Despite all these differences, there was no attempt to study possible effects of these factors on growth and development of antler cells *in vitro,* although they all may be of high importance.

Another possible factor influencing the antler cells *in vitro* is the individuality of each animal, i.e. inter-individual differences among the cells from different animals. This is important, since inter-individual variation of antler growth and size plays a significant role in the social behaviour and reproductive success of the deer species [24], [25]. Inter-individual differences are also an often-described feature of mesenchymal stem cells [13], [20], [26], [27]. However, individuality has not been explicitly taken into account in any of the *in vitro* experiments on antler cells [4], [5], [9]–[11], [23].

In the presented study we investigated the significance of factors affecting the proliferation potential of antler cells from three individual red deer males (*Cervus elaphus*). Samples were taken from the regenerating antler tip during the most rapid growth phase of antlers on the 30^th^ and 60^th^ day of the antlers re-growth after previous antlers were cast [2]. The cell proliferation was measured by incorporation of ^3^H thymidine in primary cultures or in the second passage cultures and cultivated with 10% or 1% of FCS. We hypothesized, that inter-individual differences will show up in all culture conditions, identically in both sampling days, but may vary with changing passage and percentage of FCS.

## Materials and Methods

### Antler tissue

All experiments were conducted under the approval of the Institute of Animal Science and Central Commission for Animal Welfare (Ministry of Agriculture of the Czech Republic) Committee (protocol code 26847/2006-17210).

**Figure 1:**
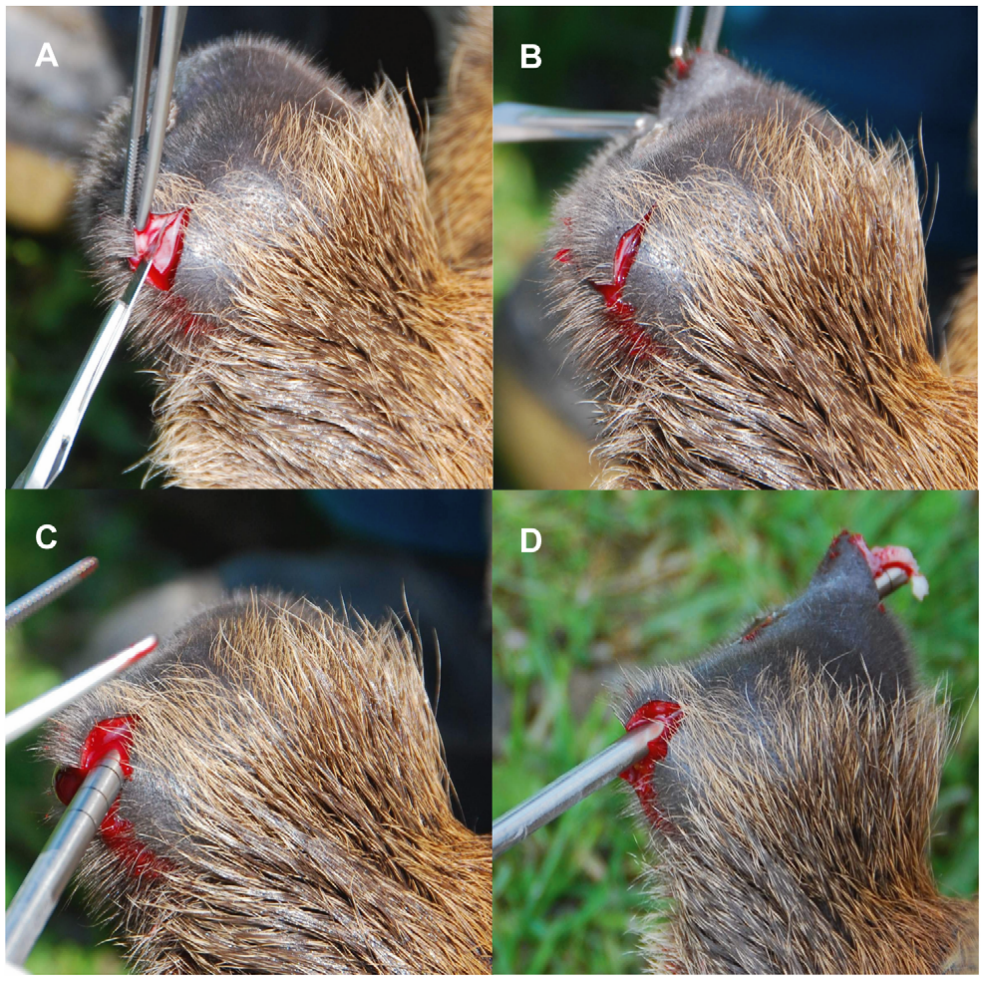
Tissue sampling. Example of tissue sampling from anesthetized animals using a sterile trephine punch.

Three three-year old farmed red deer males were fully immobilized with 30 ml intramuscularly injected Hellabrunn mixture [187.5 mg Xylazine (Bioveta, Prague, Czech Republic) +150 mg Ketamine (Bioveta, Prague, Czech Republic) in 1 ml, used 0,2 ml/10 kg of life weight] by a veterinarian in a crush. Subsequently the growing tips of regenerating antlers were superficially cleaned with a disinfection agent Spitaderm (Ecolab, 509-302056). Approximately 0.5 – 1.0 cm from the antler tip where the growth zone was reported [28], [29] a biopsy was taken. This zone is considered as an abundant source of cells for *in vitro* studies [1], [30]. The biopsies were performed on the 30^th^ and the 60^th^ day after the initiation of a new antler growth. The epidermis and the dermis were cut with a scalpel in a “V” shape and were diffracted to enable the underlying tissue for the biopsy. This was performed with a sterile trephine punch (Ø6 mm, Eickemeyer, 184905) (Fig. 1.). The obtained tissue was immediately put into a sterile tube containing “manipulation medium” DMEM/F12 containing 1% Insulin-Transferin-Selenium Supplements (ITS), 1% Antibiotic Antimycotic solution, 0,1% Gentamycin and 5% FCS (all reagents were from Gibco/Invitrogen, Prague, Czech Republic).

### Cell isolation and culture conditions

The tissue was processed immediately (within 30 min.) after the biopsy. The cells were acquired by a combination of two methods as described by Sadighi et al. [9] and Faucheux et al. [23]. Briefly, the tissue was washed with Hanks Balanced Salt Solution containing 1% Antibiotic Antimycotic solution and 5% FCS. Specimens were mechanically minced into pieces approximately 0.5–1 mm^3^ in size using a sterile scalpel, under aseptic conditions in a laminar flow hood, washed again and incubated in “standard medium” DMEM/F12 1:1 containing 1% Penstrep, 1% ITS and 0,1% Gentamycin with 200 U/ml Type II Collagenase (Gibco/Invitrogen, Prague, Czech Republic) for 4 hours at 37°C. Samples were continuously vortexed every 20 min. Obtained cells were immediately sieved and seeded into experiment as primary culture (60^th^ day after antler casting) or cultivated in the density of 4–5.10^4^ cells per cm^2^ until reaching confluence and second passage (within 6–8 days) was seeded into the experiments (30^th^ and 60^th^ day after antler casting). In both cases, cells were seeded in 48-well plates (Nunc) at a density of 4.10^4^ cells per well, followed by a 24-hour-cultivation in 1% FCS and by a 2×24-hour-cultivation in 1% or 10% FCS, all in a triplicate way. The cells were incubated at 37°C in 5% CO_2_ and 95% air.

### Cell proliferation essay

To determine the cell proliferation potential, 16 hours before the termination of incubation ^3^H thymidine (Methyl-^3^H thymidine, s. a. 6–7 Ci/mmol, ICN, USA) was added in the final concentration of 1 µCi/ml into each well. The DNA synthesis was measured by incorporation of ^3^H thymidine using the technique of TCA precipitation and liquid scintillation counting as described in Vacková et al. [31].

### Statistics

Associations between antler cells proliferation, two antler growth stages (30, N = 12 and 60, N = 36, days after the antler casting), individual males (A, N = 18; B, N = 18 and C, N = 18), the passage (primary culture, N = 18 and passaged cells, N = 36) and the percentage of FCS (1%, N =  27 and 10%, N = 27) were tested using multivariate General Linear Mixed Model (GLMM) with incorporation of ^3^H thymidine as the dependent variable and the variables described above as fixed effects. To account for the repeated measures on the same individuals, all analyses were performed using mixed model analysis with individual deer in an interaction with the passage as a random factor, using PROC MIXED (SAS, version 9.1). The significance of each fixed effect in the mixed GLMM was assessed by the F-test, on sequential dropping of the least significant effect, starting with a full model. In unbalanced designs with more than one effect, the arithmetic mean for a group may not accurately reflect a response for that group, since it does not take other effects into account. Therefore, we used least-squares-means (LSMEANs) instead. LSMEANs are, in effect, within-group means appropriately adjusted for the other effects in the model. LSMEANs were computed for each class and differences between classes were tested by t-test. For multiple comparisons we used the Tukey-Kramer adjustment.

## Results

**Figure 2:**
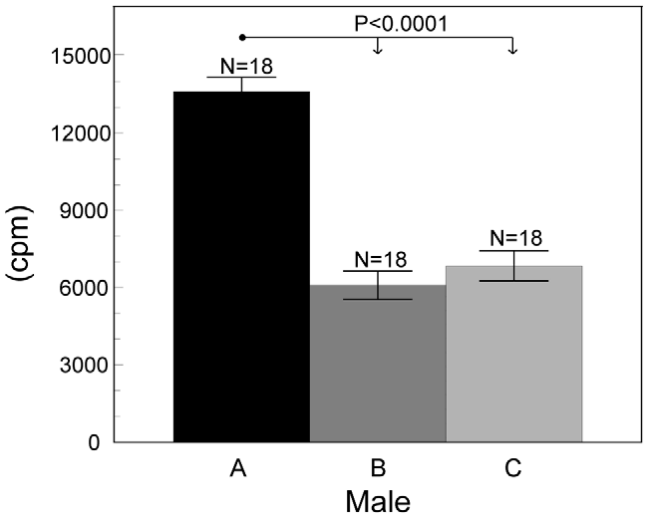
Effect of the individual males on the antler cell proliferation. Incorporation of ^3^H thymidine in antler cells (least square means ± S.E.) according to the individual males (A, B, C). All other factors were statistically eliminated.

**Figure 3:**
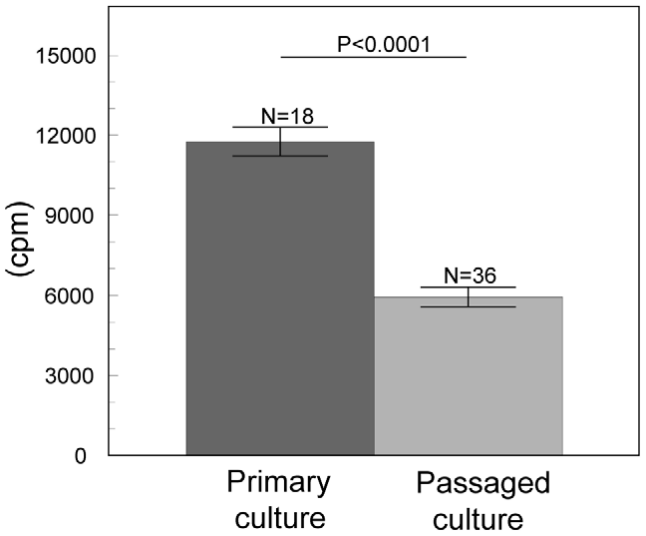
Effect of the passage on the antler cell proliferation. Incorporation of ^3^H thymidine in antler cells (least square means ± S.E.) according to the passage (primary culture, passaged culture – 2^nd^ passage). All other factors were statistically eliminated.

**Figure 4:**
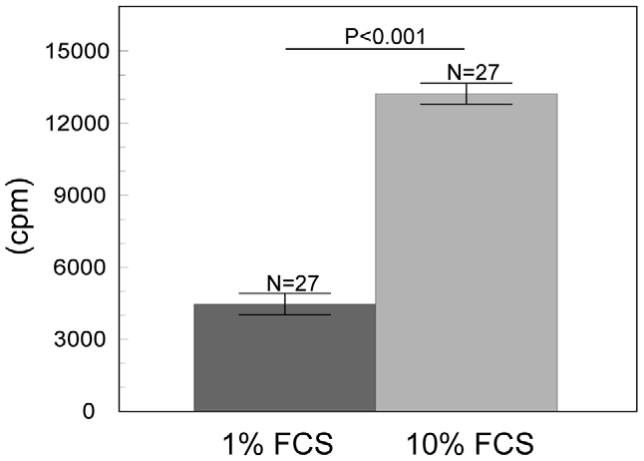
Effect of the FCS on the antler cell proliferation. Incorporation of ^3^H thymidine in antler cells (least square means ± S.E.) according to FCS percentage. All other factors were statistically eliminated.

**Figure 5:**
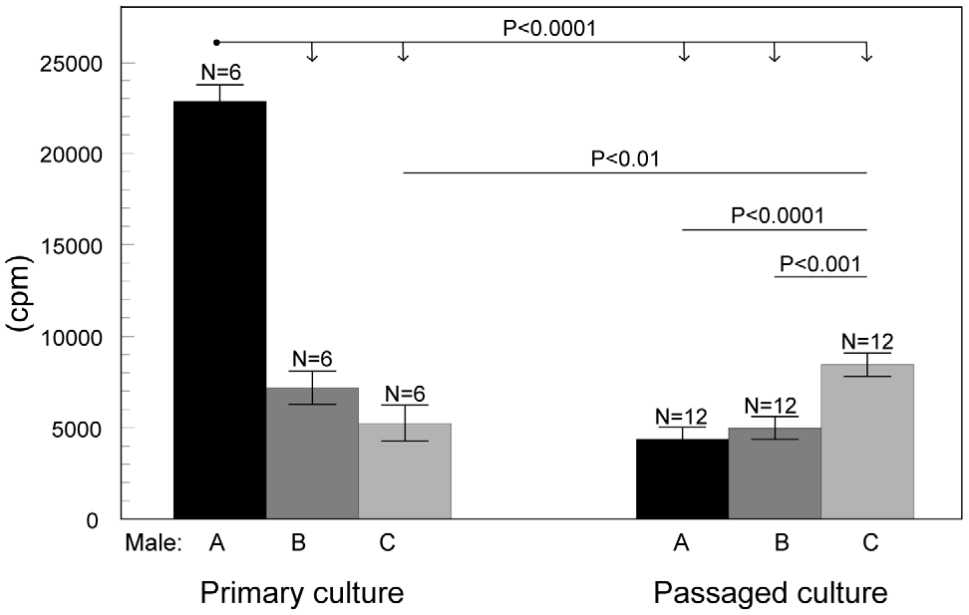
Effect of the interaction between individual males and passage on the antler cell proliferation. Incorporation of ^3^H thymidine in antler cells (least square means ± S.E.) - the interaction between individual males (A, B, C) and passage (primary culture, passaged culture – 2^nd^ passage). All other factors were statistically eliminated.

Proliferation of growing antler cells depended on all investigated factors (such as male individuality, passage and percentage of FCS) but not on the stage of antler growth. The final GLMM model contained fixed effects of the male individuality (F_(2, 46)_ = 56.11, P<0.0001 Fig. 2), passage (F_(1, 46)_  = 80.53, P<0.0001 Fig. 3), percentage of FCS (F_(1, 46)_  = 210.65, P<0.0001 Fig. 4) and an interaction between individual males and cell passage (F_(2, 46)_  = 101.37, P<0.0001 Fig. 5). The proliferation of antler cells was highly affected by male individuality. As predicted, the intensity of proliferation of particular individuals was identical between the two antler growth stages, since no significant effect of antler growth stage was confirmed. Higher percentage of FCS (10%) emphasized the inter-individual differences among the males apparent in the 1% FCS, while passage changed the proportion of the proliferative intensity among the males (Fig.4). Moreover cells of particular individuals cultivated as a primary culture, without passaging, reacted with significantly higher intensity than cells after passage. Not surprisingly 10% of FCS stimulated cell proliferation more than 1% of FCS.

## Discussion

In agreement with our predictions, the results clearly show that the factors such as 1) male individuality, 2) whether the antler cells were passaged or not and 3) concentration of FCS in the cultivation medium significantly affected antler cell proliferation *in vitro*. The only tested factor, which did not influence the antler cell proliferation, was the stage of antler growth.

Our work differs from the previously published works by sampling on both the 30^th^ and the 60^th^ day after antler casting, from the same individual. In this way we obtained and compared cells twice during the antler growth phase. However the time interval between the two growth stages on the 30^th^ and 60^th^ day was probably not sufficient to demonstrate any significant differences and samplings from earlier stages would be needed to point out potential differences in the proliferation intensity of antler cells.

Over the last years, a stem cell based origin of antlers was discussed and confirmed [32]–[36] and stem cells were found and isolated from regenerating antlers [3]. These MSC positive to surface antigen STRO-1 were shown by Rolf et al. [3] to differentiate into the “mesenchymal stem cell golden standard” - osteogenic, adipogenic and chondrogenic lineages. MSC are of great biomedical promise and a vast research interest is dedicated to their biology [37]. Recently we have shown that considerable amounts of MSC (up to 38%) can be isolated from mixed antler cell cultures [22]. This allows us to compare some of the MSC culture characteristics to our antler cell cultures.

We found a highly significant effect of male individuality on proliferation potential of antler cells. Similarly, a great inter-individual variability has been reported for ovine mesenchymal stem cell colonies [13] and for rabbits in the proliferative behaviour of the bone-marrow mesenchymal progenitor cells [20]. Ciapetia et al. [26] reported highly variable osteogenic potential in femur-derived human MSC among patients, unrelated to sex or age. In another study, Riekstina et al. [27] found very high inter-individual proliferation variability in skin-derived mesenchymal stem cell and their response to fibroblast growth factor-2, which after 3 days in culture overrode the effect of the growth factor and a generalized estimate of its effect was not possible.

In the present study, the rate of antler cell proliferation was significantly higher in 10% FCS than in 1% FCS in both primary and passaged culture. Such a result is not particularly surprising considering that cells in general proliferate more intensive in 10% FCS than in 1% FCS [19], [38]. Berg et al. [39] reported that 81.9% of undifferentiated antlerogenic periosteum cells proliferate in 10% FCS whereas just 1.4% of cells cultivated in 0.5% FCS, which is similar to our observation.

Using 10% FCS may also lead to a reduced or changed expression of biochemical markers. Pradel et al. [19] did not find any significant effect of 10% FCS on the human osteoblast-like cells morphology between primary and second passage culture. On the other hand Pochampally et al. [40] reported, that the human mesenchymal stem cells (hMSC) cultivated in 10% FCS differentiate and change their superficial expression markers more quickly, while cells cultivated without serum express the markers of undifferentiated cells much longer. Yokoyama et al. [41] demonstrated that components of FCS could stimulate hMSC differentiation to chondrocytes while a lower concentration could decrease this differentiation. This is in contrast to Price et al. [42], who stated that unlike mesenchymal cells from a developing limb, the antler cells in the culture spread out, form monolayers and do not initiate chondrogenesis. Nevertheless, previously mentioned studies have indicated that independently of performing the experiments in serum free conditions, the precultivation of antler cells in 10% FCS [9]–[11] may cause the cells to react differently from cells of primary culture or cells *in vivo/in situ.* This could explain the differences among results of various studies of hormonal and grow factors influence on antler cell proliferation [1], [4], [5], [10], [11]. Experiments using FCS during precultivation should therefore be interpreted with caution and it seems more appropriate to simulate *in vivo* conditions by primary cultures with only shorter exposure to FCS, as it was done by Faucheux et al. [23].

On the other hand, there are interesting indications by Patel et al. [17] on pulpal tissue, where the expression of markers regarded as being indicative of odontoblasts are considerably under-represented in primary culture compared to pulpal tissue. Hence cells immediately isolated and passaged no longer accurately represent intact pulpal tissue. They explain this due to either loss of specific cell populations as a result of the dissociation and adhesion processes or transcriptional changes within the isolated cells due to altered environmental conditions. In the same study continued cultures demonstrated more pronounced differences, which may in their opinion represent cellular adaptation and/or selection for a particular cell population with enhanced ability to thrive on tissue culture plastic. Indeed, in agreement with Patel's' study, Uchida et al. [16] showed that primary culture and second passage of rat mesenchymal bone marrow cells differ radically in the proportion of three detected cell populations.

As indicated above, during passaging, which is often performed to obtain sufficient numbers of cells, the cells change their morphology, capability to multiply and differentiate, and their gene expression changes dramatically [14]–[19]. A variation of the gene expression during passaging was confirmed also in cell lines [43] and the authors warn that even comparisons of analyses of cell line cultures carrying the same name may be dangerous.

In conclusion most *in vitro* hormonal and growth factor experiments with cultivated pedicle and antler cells have so far been performed after two passages [4], [5], [9]–[11]. Li et al. [5] stated that the reaction of such cells might represent the *in vivo* situation. This however is notably in contrast to recent literature and our results which show, that primary culture without any passaging and long term FCS treatment may be more related to the *in vivo* conditions. We suggest, that before all possible main factors affecting antler cells proliferation *in vitro* will be satisfactorily investigated, results of *in vitro* studies focused on hormonal regulation of antler growth [1], [4], [5], [9]–[11] should be taken with increased caution.
